# Analysis of a safe utilization algorithm for retired power batteries from new energy vehicles

**DOI:** 10.1016/j.heliyon.2024.e23994

**Published:** 2024-01-04

**Authors:** Daobao Luo, Jianguo Han, Xin Hu

**Affiliations:** School of Automotive and Transportation, Henan Polytechnic, Zhengzhou, 450046, China

**Keywords:** New energy vehicle, Lithium-ion battery, Genetic algorithm, Filtering algorithm, State detection

## Abstract

The graded utilization of waste batteries has gained research significance due to recent reports of new energy vehicle lithium-ion batteries exploding whilst awaiting recycling or in end-of-life storage. In this study, we innovatively selected battery performance parameters such as the internal resistance, charge and discharge rate, and current maximum available capacity to evaluate the safety of retired power batteries from the perspective of inducing thermal runaway. A fractional calculus theory was then introduced, and the fractional second-order resistance as well as a capacitance model and an adaptive genetic algorithm were established for the identification of the parameters. An improved dual-scale filtering algorithm was generated, which combined the extended Kalman filter algorithm and the unscented Kalman filter algorithm to improve the accuracy of the parameter estimation. The final test outcomes indicated that the equivalent circuit model optimized by incorporating multiple filtering algorithms had error rates of 1.87 %, 1.65 %, and 1.27 % for the state of charge of the battery in three different operating condition testbeds, with average errors of 0.62 %, 0.69 %, and 0.59 %, respectively. When an initial experimental platform was constructed for the detection of the parameters, the voltage error quickly stabilized to within 0.03 V. It also displayed many advantages of data detection and calculation, such as faster convergence, faster tracking, and the highest result accuracy when compared with the battery model using other algorithms. This experiment highlighted that a fractional second-order resistive–capacitive circuit equivalent battery state detection model incorporating various filtering algorithms has practicality and feasibility.

## 1 Introduction

By 2022, there were approximately 1.5 billion vehicles in the world. Many vehicles emit exhaust gases that have a serious impact on the environment as well as global climate change. To address the problem of global warming caused by excessive carbon dioxide emissions, new energy vehicles have become the first choice in the automobile market. Governments of various countries have introduced a series of encouraging policies such as tax and fee reductions to promote the development of new energy vehicles. Consequently, new energy vehicles have experienced a rapid growth in recent years [1]. However, the battery technology of new energy vehicles requires further optimization. At present, the life of power batteries is generally between 5 and 8 years; thus, energy storage batteries used in the early stages of new energy vehicle popularity now require recycling or scrapping. These waste batteries contain a variety of heavy metals and toxic electrolytes as well as other pollutants, which pose a major threat to the ecological environment and human health. The requirement to recycle waste batteries will only exponentially increase; therefore, the reasonable treatment and effective utilization of these waste batteries are crucial [2]. Although used batteries are no longer suitable for high-energy consumption devices such as cars, they retain a certain practical value for low-energy consumption devices such as charging computer mice. The fast-gradient classification of used batteries is important; it also has significant potential for commercialization. To realize the fast-gradient classification of waste batteries of different quality, accurate internal data of the battery are required for various parameters; this is also convenient for battery classification and utilization. In this context, researchers have attempted to establish an equivalent circuit battery model based on a two-stage resistance–capacitor circuit. This model combines an adaptive genetic algorithm with dual-scale filtering algorithms to improve the timeliness and accuracy of the battery state detection model. This may also help to solve the problems of waste battery management and effective utilization.

In this study, we explore and analyze the technology from four aspects. First, we discuss and summarize the current research related to the safety detection and identification of the parameters and states of energy storage of lithium-ion batteries from NEVs. Second, we present a computational analysis of the integrated application of an EC model, a genetic algorithm, and a Kalman filter algorithm (KFA), including the construction of battery parameters and a state detection system. Third, we explain the experimental verification and data analysis of the battery parameters and state detection model. Finally, we present a comprehensive overview of the whole paper and a reflective summary of the shortcomings.

## 2 Related work

The recycling of used batteries requires not only the detection of internal parameters, but also the real-time detection of the stability and safety of batteries under continuous charge and discharge states. Peng et al. proposed an improved dual-traceless Kalman filtering algorithm based on the adaptive system noise covariance and measurement noise covariance matching methods along with traceless Kalman and adaptive Kalman filtering algorithms to improve the evaluation accuracy and stability of the system [3]. Li et al. addressed the problem of how to accurately predict the battery charge state by building a new BM in view of the Davinin EC and an identification algorithm. This produced a unique method that improved the accuracy and effectiveness of battery charge state predictions [4]. A convolutional neural network combination was proposed for a thermal runaway prediction model that could predict abnormal heating. After an actual electric vehicle operation verification, the model could predict abnormal battery heating 27 min in advance with an average error (AE) of 0.28 %, enhancing the thermal runaway prediction and extending the prediction time [5]. Fernandez et al. proposed an improved adaptive dual-extended KFA for issues such as battery health state assessments. The extended KFA combined with an EC model and an online parameter identification algorithm improved the convergence and traceability of battery health state data measurements [6].

Schmid et al. presented a new fault diagnosis algorithm based on an electrothermal model for the structural analysis of reconfigurable battery systems to improve the safety, reliability, and lifetime performance of large battery packs. This also improved the proactiveness and economy of system isolations from faults [7]. Lee et al. addressed battery storage system issues that could be affected by various factors and that could become unstable. They constructed a battery data (BD) trust framework using deep learning algorithms as well as battery sensor and communication data. This framework was then combined with convolutional neural networks to propose a BD detection and classification model to improve the overall stability of battery energy storage systems [8]. Lipu et al. addressed the problem of how to accurately estimate the battery charge state. They proposed a modified random forest regression algorithm based on a differential search optimization machine learning method to improve predictions [9]. Wang et al. proposed an anti-noise adaptive long short-term memory neural network based on an improved double closed-loop observation modeling strategy to analyze the remaining useful life of power batteries and energy storage system performance evaluations, thereby improving the prediction accuracy of the remaining useful life [10]. Fernandez et al. proposed an improved iterative calculation method based on composite equivalent modeling and a new concatenated Kalman filter algorithm to improve the charging state prediction accuracy of lithium-ion battery packs [11].

Although the improved double unscented Kalman filter algorithm of Peng et al. improved the battery assessment accuracy and system stability, it was limited by the assumptions of the Kalman filter algorithm. It may not perform well for nonlinear systems with complex noise. A dual-scale filter algorithm, in contrast, can manage systems with nonlinear and non-Gaussian properties more effectively; this approach may be more advantageous in certain cases. Although the battery charge state prediction method of Li et al. improved the accuracy and effectiveness of the battery charge state, there was the potential for errors in the construction of the battery model. A dual-scale filter algorithm can estimate the system state by optimizing the measured data without relying on the exact model. The thermal runaway prediction model of Sauer et al. was remarkably successful in predicting abnormal battery heat in advance, but this may have been limited by the availability of specific conditions and data. A dual-scale filter algorithm does not need to rely on specific data and can be adapted to estimate battery states in different situations. Although the health status assessment method of Fernandez et al. improved the estimation capacity of battery health statuses, it may require other parameters and more accurate models in certain cases. A dual-scale filter algorithm can work with larger data and does not require much a priori information. The fault diagnosis algorithm proposed by Schmid et al. improved the safety and lifetime efficacy of large battery packs, but it may require complex electrothermal models and sensor placements. A dual-scale filter algorithm can be easily integrated into different types of battery systems without the need for complex models. The battery data trust framework of Lee et al. improved the stability of battery energy storage systems, but it may require a large amount of data and complex deep learning models. A two-scale filter algorithm can provide reliable state estimations with limited data. Although the machine learning approach used with the battery charge state estimation method of Lipu et al. improved the battery charge state prediction accuracy, it may require a large amount of training data and computational resources. A double-scale filter algorithm does not require large-scale training; therefore, it may be more suitable for real-time applications. The improved adaptive long short-term memory neural network proposed by Wang et al. improved the remaining useful life prediction accuracy, but deep learning models usually require a large amount of data for training; this may be limited by the data in practical applications. Neural networks are often less interpretable; it can be difficult to understand how the model formulates its predictions. This method may have significant requirements for the adjustment and tuning of the model parameters, especially the requirement to retrain or fine-tune the model for different types of batteries. This may increase the difficulty of its engineering implementation. The iterative computational approach posited by Fernandez et al. relied on composite equivalent modeling and novel filtering algorithms; specific details may vary across battery types and application scenarios, requiring a customized design and implementation. Iterative calculation methods may require greater computational resources and time, especially for applications with significant real-time requirements; thus, they may not be suitable. Compared with certain deep learning methods, a dual-scale filter algorithm does not require a large-scale training dataset. Thus, reliable state estimation results may be obtained with limited data. A dual-scale filter algorithm is generally suitable for online state estimations that do not require offline training or batch calculations, increasing its operation in practice.

The advantage of a dual-scale filter algorithm lies in its generality and robustness, which can be applied to various battery types and state estimation problems under different operating conditions without extensive prior information and complex models. An equivalent battery state joint detection model of fractional second-order resistor–capacitor circuits using multiple filtering algorithms is innovative and could improve the accuracy of battery charge state detections under the influence of multiple factors.

## 3 Design and implementation of a parameter diagnosis model for lithium-ion batteries to be recovered for energy storage

Unlike a traditional single-algorithm BM, the design concept of using multiple algorithms to consider as many influencing factors as possible is innovative. The design and implementation of a model is particularly important to ensure that the BM can be continuously optimized. This section analyzes the construction of a real-time battery state detection model based on the internal parameters of the battery.

### 3.1 Fractional-order model and genetic algorithm research

**Fig. 1:**
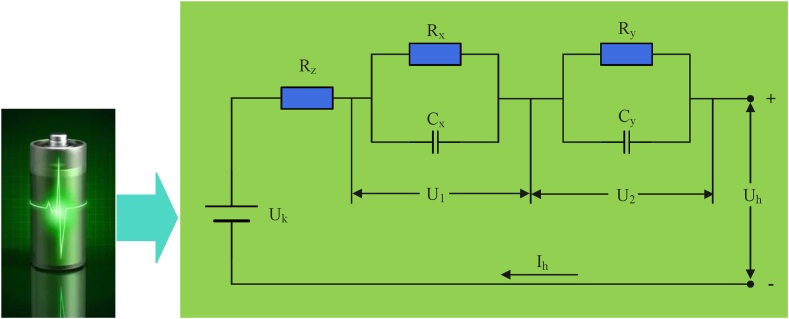
Two-stage RC EC diagram.

The power battery of an NEV is a complex energy storage system and its performance and characteristics may change over time. It is difficult to accurately capture such changes using a traditional integer-order model. A fractional-order model can describe the nonlinear and time-varying characteristics of a battery more effectively, including the complex chemical reactions and electrochemical processes inside the battery. It can also help to predict the state and performance of the battery more accurately. To analyze the internal operation principle of a battery, a battery model is used to express the internal characteristics of the battery using a circuit equation. Among the many battery models, the equivalent model of a two-stage RC fractional circuit composed of two resistor–capacitor (RC) circuits has many advantages, including easy topology, easy mathematical expression, easy parameter reading, and high precision [12]. Such a circuit is presented in Fig. 1.

Fig. 1 illustrates that the ohmic internal resistance (IR) is expressed by the ohmic resistance, the electrochemical polarization (EP) IR is expressed by the electrode resistance, the EP capacitance is expressed by the electrode capacitance, the concentration polarization resistance is expressed by the differential resistance, and the concentration polarization capacitance is expressed by the differential capacitance. In this model, the first RC ring is formed by the electrode resistance R_1x_ and the electrode capacitance C_x_ in parallel, the second RC2 ring is formed by the differential polarization resistance R_y_ and the differential polarization capacitance Cy in parallel, and the ohmic resistance Rz is connected in a series in the circuit. Using Kirchhoff's current law as the base equation, the above two-segment RC EC differential equation can be obtained, as shown in Eq. (1).

(1) $$\left\{ \begin{array}{l} dU_{1}/d_{t}=-U_{1}/R_{x}C_{x}+I_{h}/C_{x}\\ dU_{2}/d_{t}=-U_{2}/R_{y}C_{y}+I_{h}/C_{y}\\ U_{h}=U_{k}-I_{h}R_{z}-U_{1}-U_{2} \end{array}\right.$$

In Eq. (1), d represents the variable coefficient, $U_{1}$ is the voltage of the RC1 ring, $U_{2}$ is the voltage of the RC2 ring, $U_{k}$ represents the potential difference between the two terminals when the battery is not charged or discharged, $U_{h}$ represents the voltage in the positive and negative terminals, and $I_{h}$ represents the load of the current. As the determination of the remaining battery charge is related to the accuracy of determining the rated capacity in different states, it is essential to use the state of charge (SOC). The time integration algorithm is presented in Eq. (2) [13].

(2) $$SOC\left(t\right)=SOC_{0}-\frac{\int_{0}^{t}\eta I_{h}d_{t}}{Q_{e}}$$

In Eq. (2), SOC(t) represents the remaining battery charge at a dynamic time, $SOC_{0}$ represents the initial charge when the time is 0, η represents the Coulomb efficiency, and $Q_{e}$ represents the maximum available battery charge. The relevant equation is derived from the SOC algorithm, as shown in Eq. (3).

(3) $$\left[\begin{array}{l} dSOC/d_{t}\\ dU_{1}/d_{t}\\ dU_{2}/d_{t} \end{array}\right]=\left[\begin{array}{ccc} 1 & 0 & 0\\ 0 & -1/R_{x}C_{x} & 0\\ 0 & 0 & -1/R_{y}C_{y} \end{array}\right]\left[\begin{array}{l} SOC\\ U_{1}\\ U_{2} \end{array}\right]+\left[\begin{array}{l} -1/Q_{e}\\ 1/C_{x}\\ 1/C_{y} \end{array}\right]I_{h}$$

In Eq. (3), SOC represents the remaining charge in the current state. As electricity is easily affected by the surrounding environment and its fundamental algorithm follows the fractional-order calculus model, an EC model can be established by the fractional-order calculus definition to describe the internal real-time performance. The differential definition is shown in Eq. (4).

(4) BtαT0f(t)=limTs→0Ts−α∑i=0[t/Ts−T0/Ts]ωiαf(t−iTs)

In Eq. (4), T0 is the initial value of the time, α is the rank order, $B_{t}^{\alpha }$ is the fractional-order operator under the influence of variable coefficient t, f(t) is the expression of the variable coefficients as a function of t, $T_{s}$ is the charge length of the model for charge sampling, [] is the data integration with the floating points removed, α and i both represent Newton binomial coefficients, and $\omega_{i}^{\alpha }$ represents the polynomial coefficients. The polynomial coefficients are derived as indicated in Eq. (5).

(5) $$\omega_{0}^{\alpha }=1,\omega_{1}^{\alpha }=\left(1-\alpha /i-1/i\right)\omega_{i-1}^{\alpha };i=1,2,...N\text{.}$$

In Eq. (5), $\omega_{0}^{\alpha }$ represents the initial value. The FO capacitance, terminal voltage, current, and SO RC state space equations are combined to obtain a continuous FO SO RC equation, which is then discretized according to the definition of FO calculus to obtain the calculus equation of the FO SO RC model. This is described in Eq. (6).

(6) $$\left[\begin{array}{l} SOC_{k+1}\\ U_{k+1}^{1}\\ U_{k+1}^{2} \end{array}\right]=\left[\begin{array}{ccc} 1 & 0 & 0\\ 0 & e^{-T_{s}^{m}/R_{x}C_{x}} & 0\\ 0 & 0 & e^{-T_{s}^{n}/R_{y}C_{y}} \end{array}\right]\left[\begin{array}{l} SOC_{k}\\ U_{k}^{1}\\ U_{k}^{2} \end{array}\right]+\left[\begin{array}{l} -T_{s}/Q_{n}\\ T_{s}^{m}/C_{x}\\ T_{s}^{n}/C_{y} \end{array}\right]I_{k}-\left[\begin{array}{l} 0\\ \lim_{T_{s}\to 0}\sum_{i=1}^{N}\omega_{i}^{m}U_{k+1-i}^{1}\\ \lim_{T_{s}\to 0}\sum_{i=1}^{N}\omega_{i}^{n}U_{k+1-i}^{2} \end{array}\right]$$

In Eq. (6), k represents the differential coefficients, m represents the input parameters, and n represents the output parameters. As the equation operator is calculated based on all states of the memory history, the number of polynomial terms should be less than k to ensure excessive computation does not occur. A fractional-order model can more accurately estimate the SOC (state of charge) and SOH (state of health) of a battery to facilitate the early detection of battery problems. It can also establish a more intelligent charging and discharging strategy, improving the efficiency and life of the battery. The optimal management of batteries must consider multiple parameters and constraints such as charging and discharging strategies, temperature control, and battery health. A genetic algorithm is a powerful tool that can solve multi-parameter optimization problems and uses the best combination of battery operation strategies to maximize battery life, performance, and safety. This method can also supply comprehensive and multi-dimensional optimization results, providing new possibilities for the safe utilization of batteries. Our proposed circuit model used an adaptive genetic algorithm (AGA) to adaptively transform the genetic parameters to improve the accuracy and diversity of the data. The mutation rate (MR) plays a key role in the AGA, along with probability and the mixed crossover rate (COR) [14]. Due to the constant changes in current and voltage in the discharging state, each set of test data should have different MRs and CORs for the detection results. High-quality cells in the data should be retained and low-quality cells should be generated by mutation and interleaving to generate new cells, resulting in MR and COR expressions as presented in Eqs. (7), (8).

(7) $$MR\left(i\right)=\left\{ \begin{array}{l} K_{1}\left(f_{\max}-f\left(i\right)\right)/{(f_{\max}-f\left(i\right)}_{ave}),f\left(i\right)\geq f{\left(i\right)}_{ave}\\ K_{3},f\left(i\right)<f{\left(i\right)}_{ave} \end{array}\right.$$

In Eq. (7), MR(i) is the probability of variation under a parameter, i is the coefficient of variation, $f_{\max}$ is the maximum adaptation value of the population, $f{\left(i\right)}_{ave}$ is the average adaptation value of the population under a parameter, and K is the number of evolutions.

(8) $$COR\left(i\right)=\left\{ \begin{array}{l} K_{2}\left(f_{\max}-f\left(i\right)\right)/{(f_{\max}-f\left(i\right)}_{ave}),f\left(i\right)\geq f{\left(i\right)}_{ave}\\ K_{4},f\left(i\right)<f{\left(i\right)}_{ave} \end{array}\right.$$

**Fig. 2:**
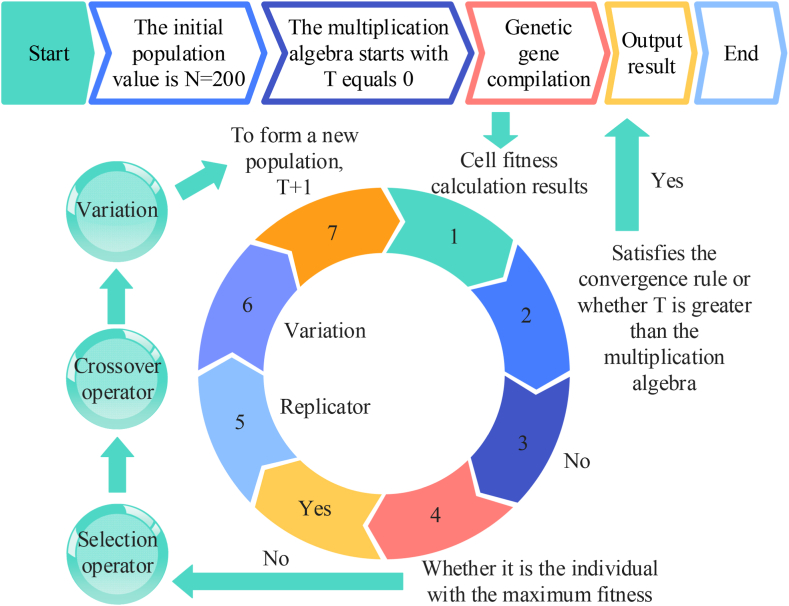
Flowchart of genetic algorithm.

In Eq. (8), COR(i) is the crossover probability under a certain parameter. The specific flow of applying an AGA algorithm with an adaptive adjustment of the dual probabilities to the SO RC model is presented in Fig. 2.

**Fig. 3:**
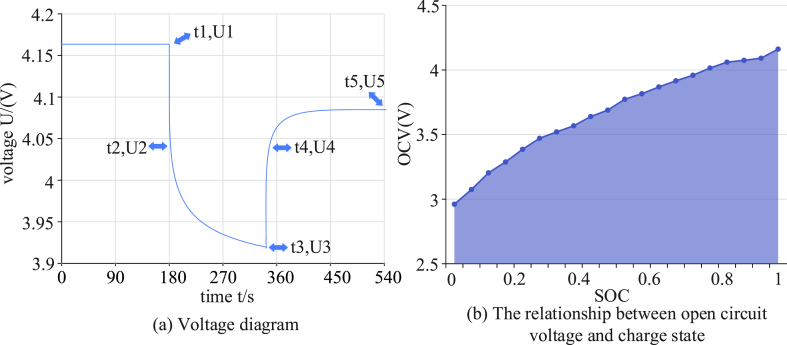
Part of a data voltage diagram and an open circuit voltage and charge state relationship diagram.

As presented in Fig. 2, to lay the foundation for the subsequent continuous reduction in the accuracy disparity of the measured data as well as the predicted data, we simulated an electrical signal based on a certain type of test bench to continuously output the pulse signal to the model to test the accuracy of the data acquisition of the SO RC circuit model under the genetic algorithm. A few of the voltage test plots from the data as well as open circuit voltage versus charge state plots are presented in Fig. 3.

Fig. 3(a) illustrates that the model detected an instantaneous change in charge at time point t1. The voltage instantaneously decreased from U1 to U2 due to the resistor blocking the current. After decreasing to a certain level due to the internal charge deviation effect, the decreasing trend gradually slowed down at time point t2 until the lowest charge state (U3) was reached at time point t3. At the end of the pulse, the voltage instantaneously rose to the level of U4 at time point t4 and then gradually returned to a slightly lower state (U5) at time point t5. This was slightly lower than before the pulse due to the continuous no-load and consumption of the charge. In Fig. 3(b), OCV indicates the potential difference between the two ends of the battery at the point of standing for a period of time without charging and discharging. The experiments tested the relationship between the voltage and the state of charge when the battery was standing and stable at a certain moment in charging and discharging. The voltage level and the state of charge were essentially proportional to the nonlinear change, providing an effective data reference to improve the accuracy. The application of the fractional-order model and a genetic algorithm in the safe utilization algorithm analysis of retired power batteries of new energy vehicles may provide more accurate, comprehensive, and innovative solutions to optimize the performance, life, and safety of batteries to promote the development of new energy vehicle technology and the sustainable utilization of waste batteries.

### 3.2 Design and implementation of a double-scale signal processor model

To improve the estimation accuracy of the algorithm model for parameters such as the maximum rated capacity of the battery, the SOC, and various types of resistance values, we introduced the extended Kalman filter (EKF) and unscented Kalman filter (UKF) algorithms. The behavior of power batteries is usually nonlinear; the nonlinear characteristics under different operating conditions are particularly obvious. Although a fractional model may describe the nonlinear characteristics of a battery more accurately, EKF and UKF can effectively account for system uncertainties such as measurement noise and process noise to describe the battery behavior more accurately. EKF and UKF can also provide high-accuracy state estimations at each time step by recursively updating the state estimate. Genetic algorithms usually involve the optimization of a group of particles or individuals limited by the number of particles. EKF and UKF based on probability density filtering methods do not require a large number of particles, so they can be estimated in high-dimensional state space. Thus, the internal state estimation of the battery is more robust. The EKF algorithm can be applied to the nonlinear model of the key data linear transformation to remove noise to obtain a higher precision estimate. This is the primary method for the nonlinear Taylor expansion equation, as presented in Eq. (9) [15].

(9) $$\left\{ \begin{array}{l} f\left(x_{k},u_{k}\right)\approx f\left({\hat{x}}_{k},u_{k}\right)+\partial f\left(x_{k},u_{k}\right)/\partial x_{k}{|}_{x_{k}={\hat{x}}_{k}}\left(x_{k}-{\hat{x}}_{k}\right)\\ g\left(x_{k},u_{k}\right)\approx g\left({\hat{x}}_{k},u_{k}\right)+\partial g\left(x_{k},u_{k}\right)/\partial x_{k}{|}_{x_{k}={\hat{x}}_{k}}\left(x_{k}-{\hat{x}}_{k}\right) \end{array}\right.$$

In Eq. (9), $f\left(x_{k},u_{k}\right)$ represents the state migration function, $x_{k}$ is the current state value (SV) of the system, $\hat{x}$ represents the optimal SV at a certain time, $u_{k}$ represents the current action at a certain time, $g\left(x_{k},u_{k}\right)$ represents the detection function at the current state, and $g\left(x_{k},u_{k}\right)$ represents the variable coefficient. The linear discrete variation of the equation is shown in Eq. (10).

(10) $$\left\{ \begin{array}{l} x_{k+1}\approx \partial f\left(x_{k},u_{k}\right)/\partial x_{k}{|}_{x_{k}={\hat{x}}_{k}}x_{k}+\left[f\left({\hat{x}}_{k},u_{k}\right)-\partial f\left(x_{k},u_{k}\right)/\partial x_{k}{|}_{x_{k}={\hat{x}}_{k}}{\hat{x}}_{k}\right]+\omega_{k}\\ y_{k}\approx \partial g\left(x_{k},u_{k}\right)/\partial x_{k}{|}_{x_{k}={\hat{x}}_{k}}x_{k}+\left[g\left({\hat{x}}_{k},u_{k}\right)-\partial g\left(x_{k},u_{k}\right)/\partial x_{k}{|}_{x_{k}={\hat{x}}_{k}}{\hat{x}}_{k}\right]+\upsilon_{k} \end{array}\right.$$

**Fig. 4:**
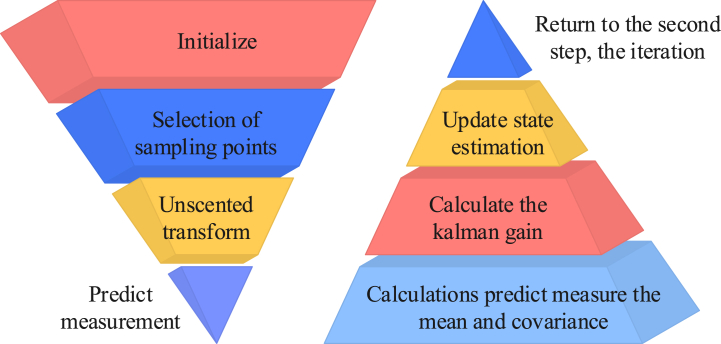
Flowchart of untraced Kalman filtering.

In Eq. (10), $y_{k}$ is the observed values of the system; both ω and υ are nonlinear real numbers. These are then applied to the Kalman standard algorithm, which is the EKF algorithm, to await further applications. Although the EKF algorithm is easier to use overall, it suffers from a heavy computational burden and large errors when targeting low-precision models with high nonlinearity. Therefore, it should be combined with the unscented Kalman filter (UKF) for the joint detection of high and low parameters. The specific flow of a UKF system for the trace-free transformation of initial data after selecting the sampling points is presented in Fig. 4 [16].

Fig. 4 illustrates that the UKF algorithm should be initialized first. This consists of initializing the mean and covariance matrices of the state estimates, the covariance matrices of the system, and the measurement of noise. The uncertainty of the current state is then represented by generating a set of sampling points from the mean and covariance matrix of the current state estimate. A nonlinear function is then used to map each sample point to obtain the predicted state and predicted measurement. After the measurement is predicted, the mean and covariance are calculated and the Kalman gain is used to update the mean and covariance of the state estimate. Finally, the above steps are iteratively repeated and the state estimation is updated by measuring the data each time to predict the state at the next moment. Although UKF has greater advantages than a standard Kalman filter (KF) and extended Kalman filter (EKF) when dealing with nonlinear systems, the local linearization of UKF may not be able to effectively approximate a real system in systems with extremely high nonlinearity, resulting in an increase in estimation errors and poor performance in highly nonlinear systems. Therefore, a fractional-order unscented Kalman filter (FOUKF) should be obtained, which improves UKF using a fractional order. The calculation of sigma points (s) is shown in Eq. (11) [17].

(11) $$\left\{ \begin{array}{l} x_{k}^{0}={\hat{x}}_{k}\\ x_{k}^{i}=x_{k}+{\left(\sqrt{\left(L+\lambda \right)P_{k-1}}\right)}_{i},i=1,2,3,...L\\ x_{k}^{i}=x_{k}-{\left(\sqrt{\left(L+\lambda \right)P_{k-1}}\right)}_{i-1},i=L+1,L+2,L+3,...2L \end{array}\right.$$

In Eq. (11), the fractional-order calculus transformation of the selected sigma points yields its system optimal valuation equation as shown in Eq. (12) and the covariance error equation as shown in Eq. (13).

(12) $${\hat{x}}_{k+1/k+1}={\hat{x}}_{k+1/k}+K_{k+1}\left(y_{k+1}-{\hat{y}}_{k+1}\right)$$

In Eq. (12), ${\hat{x}}_{k+1}$ is the optimal state at moment k+1.

(13) $$P_{k}=P_{xx}-K_{k+1}P_{xy}K_{k+1}^{T}$$

In Eq. (13), $P_{xx}$ is the covariance of the system prediction and $P_{xy}$ is the observed variance of the system prediction. FOUKF only considers the measurement error at a single time in the iterative process, which is not enough to reflect the real change of the system state; thus, the old data in the estimation process may cause cumulative interference. FOUKF is less robust in long-duration estimation tasks in the face of uncertainties, noise, and outliers. In this study, we innovatively introduced weighting factors of different times as well as a multi-information state measurement update mechanism to provide a more effective adaptation to highly nonlinear systems. The fractional-order multi-innovation unscented Kalman filter (FOUMIUKF) algorithm was improved and its system optimal valuation equation was updated, as shown in Eq. (14).

(14) $${\hat{x}}_{k+1/k+1}={\hat{x}}_{k+1/k}+K_{r,k+1}E_{r,k+1}={\hat{x}}_{k+1/k}+\sum_{i=1}^{2L}K_{i,k+1}\varepsilon_{k-i+2}$$

**Fig. 5:**
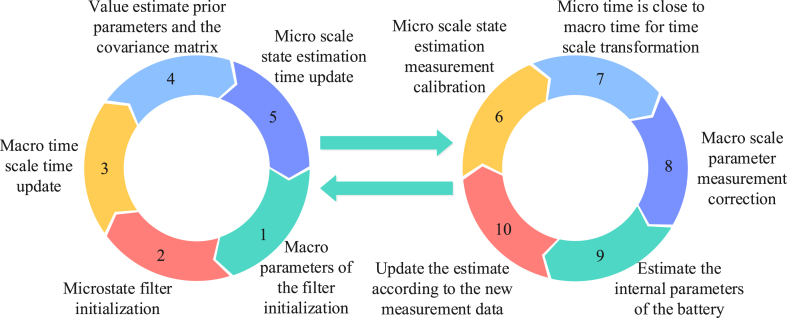
Flowchart of the dual-scale filter algorithm.

In Eq. (14), r is the length of the historical information and ε is the prediction error value at a certain state. FOUMIUKF improves FOUKF, which primarily considers measurement errors in parts of the iterative process. It introduces multi-information state measurement updates and integrates measurement error information of the previous time into the filtering process instead of only the measurement error of a single time. It can suppress the accumulated interference caused by old data and improve the accuracy of estimations. In the process of estimation, the weighting factor at different times can balance the relationship between the cumulative interference and the measurement correction according to the size of the weighting factor to improve the performance of the filter, especially in long-term estimations. In addition to global slow changes on a longer time scale, battery parameters such as voltage and current undergo nonlinear rapid changes at the instants of charging and discharging; these can be approximated as a linear system. In this paper, we combined FOUMIUKF and EKF so that the model could select the appropriate scale for estimation according to the need to meet the requirements of different application scenarios. Its process is presented in Fig. 5.

As can be seen in Fig. 5, the model divided the estimation of battery parameters and states into two levels—macro-scale and micro-scale—and improved the estimation accuracy by separately processing the macro-scale and micro-scale information. The algorithm first calculated the battery dynamic, developed on the basis of the global parameters of the nonlinear discrete mathematics expression as shown in Eq. (15).

(15) $$\left\{ \begin{array}{l} x_{k+1,l+1}=F\left(x_{k+1,l},\theta_{k+1},u_{k+1,l}\right)+\omega_{k+1,l},\theta_{k+2}=\theta_{k+1}+\rho_{k+1}\\ y_{k+1,l}=G\left(x_{k+1,l},\theta_{k+1},u_{k+1,l}\right)+v_{k+1,l} \end{array}\right.$$

In Eq. (15), F is the state transfer matrix, G is the input transition matrix, u is the input matrix, θ is the system parameter matrix, ω is the system state noise, ρ is the system parameter noise, and v is the system measurement noise. It is first initialized, then the timing currents of the filter are continuously updated in the global position to measure and correct the dynamically updated state and state prediction of the battery in the micro-position in real time to obtain the final FO SO RC equivalent BM, as indicated in Eq. (16).

(16) $$\left\{ \begin{array}{l} x_{k,l+1}=F\left(x_{k,l},\theta_{k},u_{k,l}\right)+\omega_{k,l},\theta_{k+1}=\theta_{k}+\rho_{k}\\ y_{k,l}=G\left(x_{k,l},\theta_{k},u_{k,l}\right)+v_{k,l} \end{array}\right.$$

Through a series of transformations, calculations, and the comprehensive consideration of various factors of system statuses, the observed value could be obtained. The dual-scale filter algorithm model combining FOUMIUKF and EKF was able to take full advantage of the high-accuracy estimation of FOUMIUKF and the traditional performance of EKF to achieve a more accurate state estimation. FOUMIUKF processes nonlinear and dynamic information, whereas EKF uses traditional methods to improve the overall stability of a system. FOUMIUKF, when used for dynamic weighting adjustments, can adapt to historical data with error information to minimize the effects of the cumulative error correction estimation results. This double-scale model maintains the reliability and stability of the filter algorithm and improves the estimation precision at the same time.

## 4 Model validation and result analysis

**Fig. 6:**
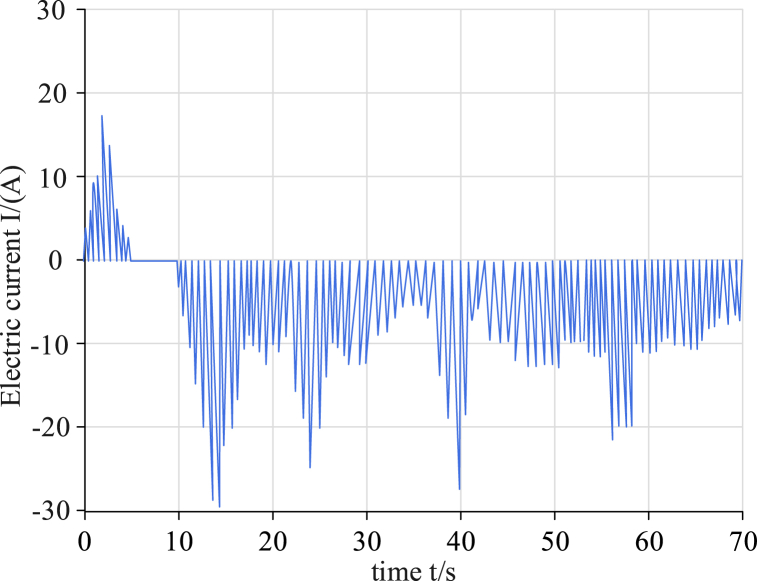
Charge change diagram of UDDS test environment.

To measure the accuracy of the dual-scale filter algorithm model, a corresponding test platform was built. The temperature control range of the incubator was from −20 °C to 70 °C and the temperature accuracy was ±0.2 °C. The internal volume was 500 L and the control mode used a PID controller. The heating power was 1000 W and the cooling power was 800 W. The capacity of the power battery pack was 50 kWh. The battery composition was 96 series, with a voltage range of 320 V–450 V. The rated capacity was 50,000 mAh. The dimensions were 1200 mm × 800 mm x 200 mm and the weight was approximately 300 kg. The battery pack shell material was aluminum alloy. The service life was 3 years of use; at this point, it was retired. The charge and discharge cycle reached 500. The core was a lithium manganate (LiMn2O4) cylindrical core. The nominal voltage was 3.7 V and the nominal capacity was 2600 mAh. The maximum charging current was 2C (5200 mA) and the maximum discharge current was 3C (7800 mA). The internal resistance was & lt and 20 mΩ. The cycle life comprised 500 charge and discharge cycles (before decommissioning). The operating temperature range was −20 °C–60 °C. The safety features were over-charge, over-discharge, and over-temperature protection. The standardized Urban Dynamometer Driving Schedule (UDDS) of the Federal Environmental Protection Agency (EPA), the New European Driving Cycle test (NEDC), the Highway Fuel Economy Test (HWFET), and three other conditions were tested using the above test platform. The accuracy of the parameter measurements of four algorithms—namely, UKF, FOUKF, FOMIUKF, and FOMIUKF + EKF—were compared using the battery model under each working condition. First, the battery was charged to a SOC of 100 % on the surface, then the temperature was reduced to a normal temperature and stabilized. Subsequently, the battery was subjected to pulse discharge and charging experiments with random charge sizes. The test current under the UDDS working condition is presented in Fig. 6.

**Fig. 7:**
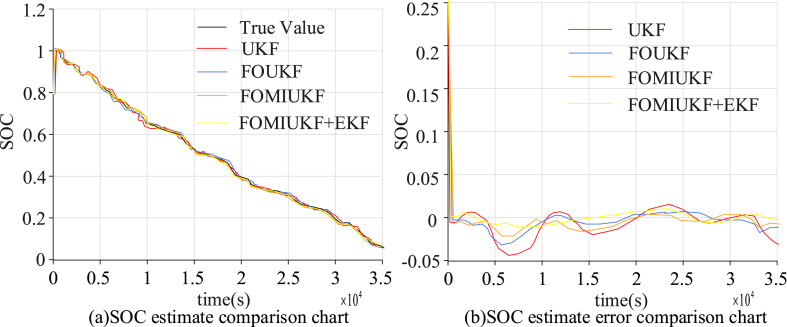
Comparison of detection results using UDDS with the four algorithms.

As UDDS simulates urban road conditions containing a large number of parking states, the charge states were also simulated as randomly changing nonlinear charge states. The results of the four algorithms for the SOC state detection and the errors are shown in Fig. 7.

**Fig. 8:**
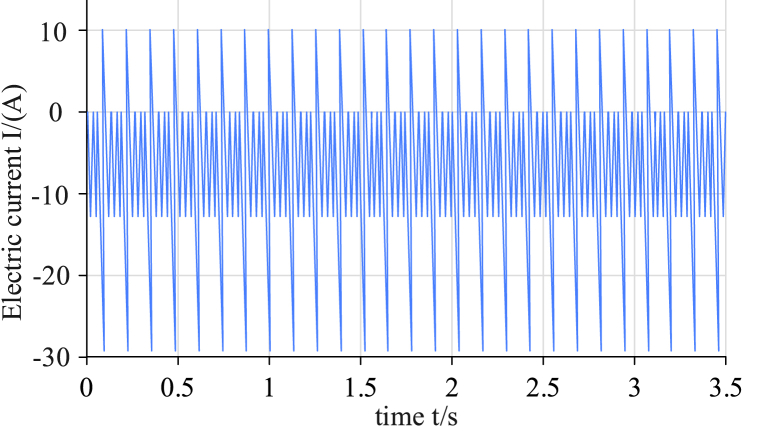
Charge variation diagram of NEDC test environment.

Fig. 7 (a) and 7 (b) show the SOC estimation results and error results of different algorithms, respectively. It can be seen that the macro perspective of the SOC state change of the UKF algorithm is not a big problem, but almost every key node has a large error, with a SOC error rate of 3.01 % and an average error of 2.12 %. The accuracy of the FOUKF algorithm improved somewhat, but a significant error remained. The SOC error rate was 2.34 % and the AE was 1.26 %. The accuracy of FOMIUKF improved when compared with the first two algorithms; the SOC error rate was only 1.96 %, with an AE of 0.89 %. FOMIUKF + EKF resulted in the best computational accuracy; the SOC error rate was reduced to 1.87 %, with an AE of 0.62 %. The second experimental environment used the NEDC conditions and the simulated current is shown in Fig. 8.

**Fig. 9:**
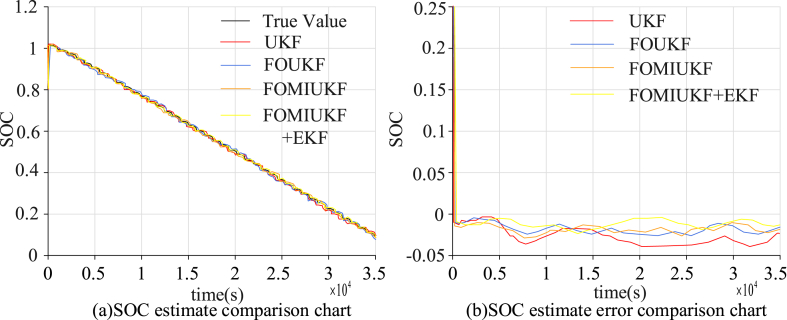
Comparison of detection results using NEDC with the four algorithms.

As presented in Fig. 8, the simulated current in this condition was a constant charge change because the majority of the environment in NEDC conditions is a low-speed or a high-speed uniform driving test. A constant value was used for acceleration. The NEDC state detection results and the error situation for the four algorithms are shown in Fig. 9.

**Fig. 10:**
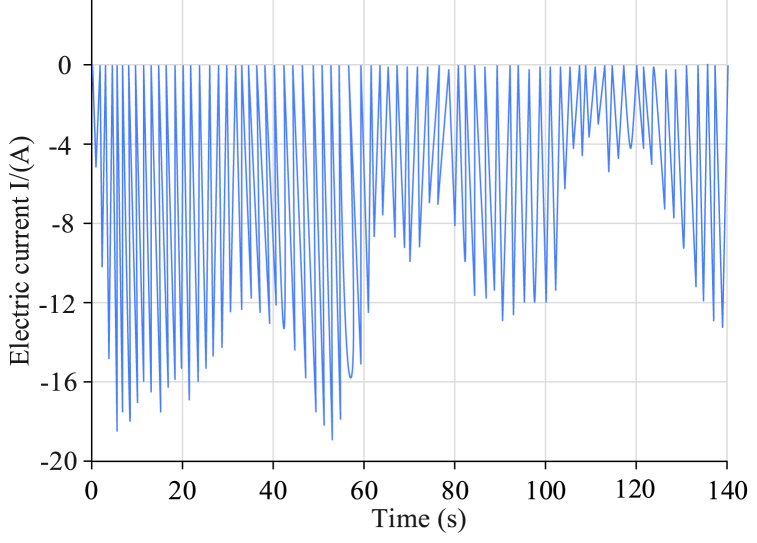
Charge variation diagram of HWFET test environment.

Fig. 9 (a) and 9 (b) show the estimation and error results of different algorithms for the NEDC state, respectively. It can be concluded that compared with other algorithms, UKF has the lowest accuracy, SOC error rate of 2.96 %, and average error of 1.89 %. The SOC error rate for the FOUKF algorithm was 2.31 % and the AE was 1.34 %. FOMIUKF was similar to the previous algorithm in terms of convergence speed, but had improved accuracy; the SOC error rate was 1.92 % and the AE was 0.84 %. FOMIUKF + EKF had the lowest error rate compared with the previous algorithms, with a SOC error rate of 1.65 % and an AE of 0.69 %. The third test environment used HWFET conditions; this charge variation is shown in Fig. 10.

**Fig. 11:**
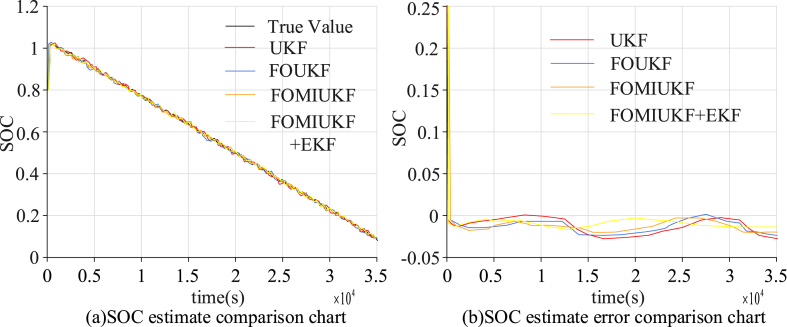
Comparison of detection results using HWFET with the four algorithms.

As presented in Fig. 10, the HWFET conditions were mainly used to test the fuel economy of the vehicle under high-speed experiments. A NEV in high-speed conditions will significantly accelerate the discharge rate of power consumption, so the simulation of the current for the high-current discharge state charge changed. The HWFET state detection results and the error situation for the four algorithms are presented in Fig. 11.

**Fig. 12:**
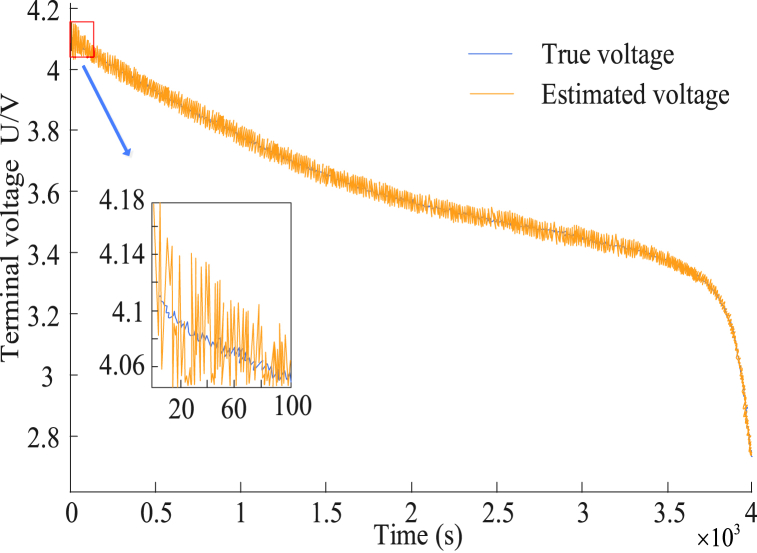
Terminal voltage recognition results.

Fig. 11 (a) and 11 (b) show the estimation and error results of different algorithms for the HWFET state, respectively. It can be observed that the four algorithms still maintain the accuracy changes under the first two operating conditions, with UKF having the highest error rate, SOC having an error rate of 2.89 %, and an average error rate of 1.33 %. The FOUKF algorithm had a SOC error rate of 1.98 % and an AE of 0.92 %. The FOMIUKF algorithm had a SOC error rate of 1.59 % and an AE of 0.74 %. The FOMIUKF + EKF algorithm could follow the actual value in real time and maintain a low error rate level under the fast-changing condition of high-speed environmental charges; the SOC error rate was 1.27 % and the AE was 0.59 %. The above experimental data proved the parameter measurement accuracy of the four algorithms (UKF, FOUKF, FOMIUKF, and FOMIUKF + EKF) in various different working conditions of a test environment, sequentially increasing the change law. FOMIUKF + EKF consisted of two filtering algorithms correcting each other in state detections. Its convergence speed in either test environment displayed certain advantages and could follow and feed back the change in the real battery state quicker than the other three algorithms. Finally, we applied the FO EC model, which incorporated the dual-scale algorithm, to the experimental initial testbed for parameter identification. The end-voltage test results are shown in Fig. 12.

**Fig. 13:**
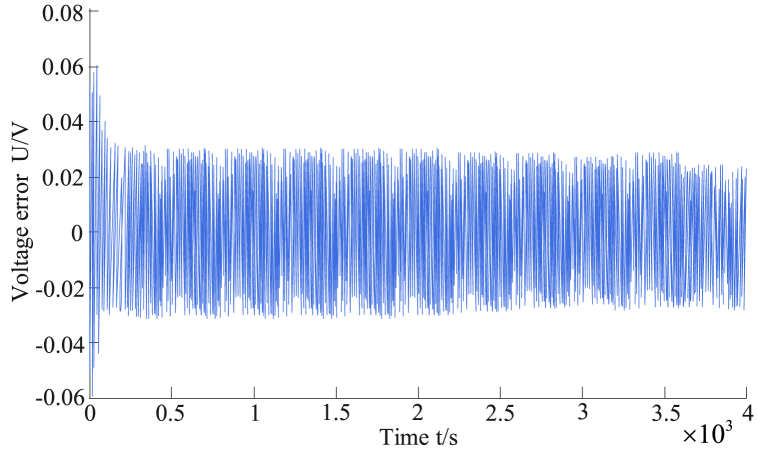
Comparison of detection errors.

Fig. 12 demonstrates that the estimated voltage produced a large error when the initial data were compared with the real voltage. The initial error was large because a large error existed in the initial input value and the actual value. The detection results gradually approached agreement with the actual parameters of the circuit. The error comparison is shown in Fig. 13.

By comparing the error values, the voltage error gradually stabilized to within 0.03 V, which was within the acceptable range for data errors, indicating that the studied BM algorithm was highly accurate. The dual-scale filter algorithm was compared with the weighted Kalman filter algorithm in Ref. [18] and with standard and unscented dual-filter algorithms in Ref. [19]. The dual-scale filter algorithm could flexibly process voltage measurement errors. By introducing multi-information state measurement updates into the micro-scale state filtering, the influence of the measurement errors on the estimations could be effectively suppressed and the estimation accuracy could be improved. The performance of the weighted Kalman filter algorithm largely depends on the selection of weights; an improper selection of weights may lead to inaccurate estimation results. The dual-filter algorithm is suitable for linear systems or the linearization of nonlinear systems. When the battery system model is complex or has strong nonlinear characteristics, these methods are usually not flexible enough to effectively manage the impact of voltage measurement errors. Adjustments and parameter fine-tuning may be required to avoid the introduction of large estimation errors. To further explore the accuracy of the proposed algorithm in the temperature and full life range, experiments were conducted on different environmental temperatures and aging stages. Among them, the ambient temperatures are −10 °C, 10 °C, 30 °C, and 50 °C, and the aging conditions are pre aging (SOH = 96.7 %), mid aging (SOH = 93.1 %), and post aging (SOH = 85.1 %). The commonly used evaluation indicators include Root Mean Square Error (RMSE), Maximum Error (MAX), and Mean Absolute Error (MAE). The SOC estimation error results of different algorithms under different environmental temperatures are shown in Table 1.

**Table 1:** SOC estimation error results of different algorithms under different environmental temperatures.

Temperature/°C	Algorithm	RMSE/%	MAE/%	MAX%
−10	FOMIUKF + EKF	2.02	1.14	2.76
	FOMIUKF	4.47	3.19	8.58
	UKF	4.76	4.02	7.57
	FOUKF	4.86	4.24	7.72
10	FOMIUKF + EKF	1.85	0.65	1.94
	FOMIUKF	4.19	2.73	9.54
	UKF	3.02	0.97	5.21
	FOUKF	3.26	1.21	5.45
30	FOMIUKF + EKF	2.07	1.07	2.46
	FOMIUKF	3.59	2.29	8.34
	UKF	2.79	2.21	4.41
	FOUKF	3.02	2.45	4.65
50	FOMIUKF + EKF	1.61	1.03	2.70
	FOMIUKF	2.12	1.39	5.97
	UKF	2.71	1.81	4.77
	FOUKF	2.95	2.05	5.01

**Fig. 14:**
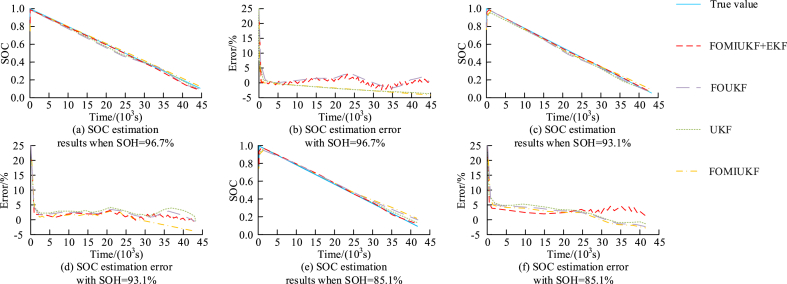
SOC estimation results and estimation error results of different algorithms under different aging states.

From Tables 1 and it can be observed that the SOC estimation MAX results of the FOMIUKF + EKF algorithm are all less than 3 %, and they can maintain good estimation accuracy in different environmental temperatures. The MAX values of the FOMIUKF algorithm at temperatures of −10 °C -50 °C are 8.58 %, 9.54 %, 8.34 %, and 5.97 %, respectively, which is much lower in accuracy than the algorithm proposed in the study. The MAX of FOUKF algorithm and UKF algorithm at 10 °C, 30 °C, and 50 °C ranges from 4341 % to 7.72 %, but at −10 °C, the maximum MAX is 7.72 %. The above results show that compared with other mainstream algorithms, the FOMIUKF + EKF proposed in the study has significant advantages at different environmental temperatures. Set the initial SOC value of the battery to 75 %, and the actual initial SOC value to 100 %. The SOC estimation results and estimation error results of different algorithms under different aging states can be obtained as shown in Fig. 14.

**Fig. 15:**
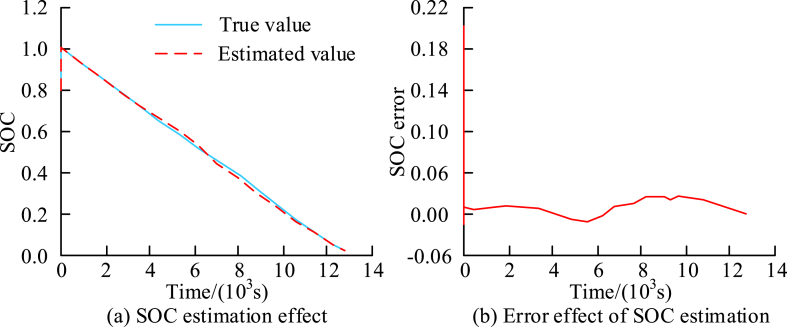
The results of FOMIUKF + EKF algorithm under actual road conditions.

Fig. 14 (a) and 14 (b) show the SOC estimation results and estimation errors for an aging state of 96.7 %, respectively. Fig. 14 (c) and 14 (d) show the SOC estimation results and estimation errors for an aging state of 93.1 %, and Fig. 14 (e) and 14 (f) show the SOC estimation results and estimation errors for an aging state of 85.1 %. From Fig. 14, it can be seen that among the estimation results of different battery aging states, the change curve corresponding to the FOMIUKF + EKF algorithm can converge to an error range of 2 % of the true SOC value in only 500 s, while other methods will continue to deepen with the degree of battery aging, especially in the later stage of battery aging, which takes an average of 1200 s to reach the optimal error range. Among the estimation error results of different battery aging states, the MAX values in the early, middle, and late stages of battery aging were 2.70 %, 2.55 %, and 2.98 %, respectively. The remaining algorithms obtained SOC estimation results with MAX values exceeding 3 %. Based on the above results, it can be concluded that the FOMIUKF + EKF algorithm proposed in the study also has excellent accuracy over the entire lifespan range. In actual road conditions, the SOC value of the battery is influenced by many factors such as driving speed and distance, so it is very important to maintain the optimal state of the battery in practical applications. To explore the effectiveness of the proposed algorithm in practical applications, experiments were conducted on the actual road conditions of high-speed driving in L city, and the corresponding results are shown in Fig. 15.

Fig. 15 (a) and 15 (b) show the SOC estimation results and estimation error results of the proposed algorithm under actual road conditions, respectively. It can be seen that the proposed algorithm can quickly follow the true values and maintain small errors throughout the entire process under actual road conditions. Its SOC MAX is 1.28 % and MAE is 0.54 %. Based on the above results, it can be concluded that temperature, aging status, and actual road conditions all have a certain impact on the battery SOC value of the proposed algorithm. However, compared with the performance of other algorithms, the FOMIUKF + EKF algorithm can effectively avoid excessive use and abuse of batteries, and has good application results.

## 5 Conclusion

Aiming at the parameter estimation problem of a large number of recycled new energy lithium-ion and other energy storage batteries, this paper first constructed a fractional second-order RC basic battery model using a second-order RC circuit and an adaptive AGA algorithm. Based on this model, a dual-scale FOMIUKF and EKF filtering algorithm using mutual corrections was proposed to improve the detection accuracy of the battery SOC state and terminal voltage as well as other parameters of the battery model. We compared experiments using three different working environments (UDDS, NEDC, and HWFET) as a test platform for the real-time detection of battery parameters and analyzed and compared the data. The SOC error rates of the battery model of the dual-scale filtering algorithm were 1.87 %, 1.65 %, and 1.27 % and the average error rates were 0.62 %, 0.69 %, and 0.59 %, respectively. The voltage error of the parameter detection on the experimental platform built at the beginning of the experiment was finally stabilized to within 0.03V. The test results revealed that in any environment, the FOMIUKF and EKF filtering algorithm using a mutual correction of the battery model followed the fastest convergence speed change state with the highest detection accuracy. In this study, we first analyzed the trigger conditions of thermal runaway in batteries and clarified the importance of parameters such as the internal resistance, charge and discharge rate, and current maximum available capacity on the battery performance. An equivalent circuit model was innovatively constructed using the fractional calculus theory. The parameters were identified using an adaptive genetic algorithm to improve the ability for the accurate estimation of battery performance. The improved dual-scale filtering algorithm innovatively integrated the fractional calculus theory and the multi-information method, improving the accuracy of the parameter estimation through the dual-scale filter mutual correction model. We proved that the equivalent battery model scheme of a fractional second-order RC circuit using a dual-scale filtering algorithm was feasible and practical. Due to limitations to the experimental time and conditions as well as the influence of other factors, our research did not cover all possible factors regarding the safety issues of power battery reuse. Further research is required to increase the depth and breadth of this study.

## Data availability statement

The data used to support the findings of this study are all in the manuscript.

## Funding statement

There is no funding in this article.

## CRediT authorship contribution statement

**Daobao Luo:** Writing – review & editing, Writing – original draft, Methodology, Conceptualization. **Jianguo Han:** Methodology, Formal analysis, Data curation. **Xin Hu:** Methodology, Formal analysis.

## Declaration of competing interest

The authors declare that they have no known competing financial interests or personal relationships that could have appeared to influence the work reported in this paper.
